# Implementing voluntary assisted dying in New South Wales correctional settings

**DOI:** 10.5694/mja2.52688

**Published:** 2025-05-26

**Authors:** Diya Ahluwalia, Leigh Haysom

**Affiliations:** ^1^ Justice Health and Forensic Mental Health Network Sydney NSW; ^2^ University of Newcastle Newcastle NSW

**Keywords:** Euthanasia, Death, Prison, Terminal care

New South Wales was the last Australian state to pass voluntary assisted dying (VAD) legislation, effective on 28 November 2023.^1^ The Justice Health and Forensic Mental Health Network (JHNSW) is responsible for the health care of people in contact with the criminal justice system in NSW (www.nsw.gov.au/health/justicehealth/) and was tasked with implementing a VAD pathway for prisoners. A steering committee was formed with the Corrective Services NSW (CSNSW; https://correctiveservices.dcj.nsw.gov.au/), the local health district, the CSNSW Inmate Consumer Referent Group, the Mental Health Review Tribunal (https://mhrt.nsw.gov.au/the‐tribunal/), the JHNSW Clinical Ethics Committee, the Victims Support Scheme and the Aboriginal Health and Medical Research Council of NSW (www.ahmrc.org.au) to create dignified and culturally safe pathways for prisoners to access VAD. The ethical and legal challenges of integrating VAD into the NSW correctional settings are highlighted below, with a hypothetical patient journey demonstrating the pathway.

## Equivalence of health care

The United Nations Standard Minimum Rules for the Treatment of Prisoners (the Nelson Mandela Rules) state that “Prisoners should enjoy the same standards of health care that are available in the community … without discrimination on the grounds of their legal status”.^2^ It is unlikely that community‐equivalent VAD in correctional settings can ever be achieved. Best practice allows terminally ill patients to have full autonomy over the timing, place and method of their death, and the loved ones who will be present; choices that are significantly curtailed for prisoners. The most community‐equivalent pathway for a terminally ill prisoner is release from custody to access VAD in the community; however, limited applications for early release are approved by the NSW State Parole Authority (https://paroleauthority.nsw.gov.au/). There have been few instances of early release from custody occurring close to the prisoner's death and these cases do not allow for a planned VAD death in the community.

## No voluntary assisted dying deaths in gaol

Feedback from the inmate referent group indicated that some terminally ill prisoners may want to die in their long term gaol to maintain connection to their “gaol family”. This was deemed unacceptable by the broader stakeholder group as it was too similar to death row, might create panic and confusion in other prisoners and leave involved staff traumatised and stigmatised. Additionally, it is a criminal offence for a prisoner to be in possession of the VAD substance in a correctional setting.^3^

The steering committee negotiated with our partner tertiary referral hospital for access to a VAD bed for the VAD substance administration step (be that patient‐ or practitioner‐administered) as close to the prisoner's chosen day of death as possible. The hospital setting allows the prisoner's loved ones to attend, noting that any visitors must undergo criminal record checks and scrutiny by CSNSW to ensure no victims are included. This process may potentially exclude significant family members. To avoid any handling of the substance on correctional grounds, the NSW Health VAD Pharmacy Service will deliver the substance directly to the hospital on the day of administration.

Shackling of prisoners is a CSNSW requirement for any escorts outside of the correctional centre; however, this was considered unacceptable for dying prisoners and would potentially interfere with patient autonomy and substance administration.^4^ Stakeholders agreed that “least restrictive practices” without any shackling would be employed for these escorts.

## Dying on country for Aboriginal patients

One third of adult prisoners in NSW are Aboriginal and Torres Strait Islander, a ten times overrepresentation.^5^ We recognise the importance of Aboriginal patients’ connection to the land and community and their choice to die on country.^6^ In consultation with CSNSW, the Aboriginal Health and Medical Research Council and Aboriginal health staff on the VAD steering committee, the agreed pathway is via a CSNSW medical escort to the most appropriate NSW health facility on country, in liaison with the NSW Health VAD Care Navigator Service and the NSW Ambulance Service. The planning for this pathway includes Sorry Business considerations with the expectation of a larger number of visitors, Sorry rituals and identifying a dedicated family contact, noting that the attendance of community members and relatives remains at the discretion of CSNSW.

## Forensic patients

The NSW Forensic Hospital is next to the Long Bay Correctional Complex and provides intensive mental health care for people determined by a Mental Health Review Tribunal (MHRT) special verdict as not guilty due to mental illness (“act proven but not criminally responsible”).^7^, ^8^ Even though terminally ill patients are unusual in this setting, it was considered important to the steering committee and the MHRT that VAD be available to eligible forensic patients. Decision‐making capacity is a fundamental requirement of VAD eligibility, and many forensic patients have capacity to provide their informed consent, noting that capacity can fluctuate, and this may need review at each VAD decision‐making step.

## Coroner inquests into all deaths in NSW custody

All deaths in custody in NSW are investigated by the NSW state coroner.^9^ Other Australian jurisdictions have made VAD deaths in custody exempt from coronial review (or review by exception); however, the NSW legislation makes no exemption.^1^ This has created anxiety for staff and made it challenging to recruit VAD practitioner roles. The strongly regulated VAD governance, eligibility criteria and NSW VAD Board approvals make further scrutiny arguably unnecessary. A coronial review or inquest with a publicly available report may also potentially breach the patient's privacy and their desire to keep their VAD death confidential. This could create additional distress for family members and victims. Accordingly, the patient is informed of the requirement for a coronial review early in the process.

## Prisoner autonomy in the correctional setting

Patient autonomy is a cornerstone principle of VAD. Informed and free decision making can be limited in a correctional setting, with institutionalised inequalities and power dynamics that can create coercive relationships between prisoners, CSNSW and NSW Health staff. The psychosocial disadvantages of prisoners can render them unable to protect their own interests and can undermine their autonomy.^10^

These vulnerabilities can be increased in the context of dying. Severely ill prisoners are extremely vulnerable, with limited or no choice about their health care providers and a forced dependency on JHNSW, CSNSW, other prisoners and family for guidance. Unaddressed pain, anxiety or depression can further compromise a prisoner's judgement. Terminal illness tends to affect older prisoners who are more likely to be on lengthier sentences, including life sentences.^11^ Length of sentence may also influence a prisoner's VAD decision.

We acknowledge these concerns and admit that prisoners will never have complete autonomy; VAD practitioners must be attuned to this disparity. We also note that coercion and vulnerability are not unique to the correctional setting and are experienced by some patients accessing VAD in the community. The VAD eligibility criteria and the carefully staged approval process have been developed to partly mitigate these concerns. Most prisoners are presumed autonomous enough to make other significant health care decisions, and so to limit their end‐of‐life choices is to abrogate their right to equivalent care.^12^

Other strategies employed to increase autonomy include ensuring that VAD consultations are conducted in private with at least one of the two attending VAD practitioners not being on the patient's usual treating team, optimising palliative care and the patient's mental health care, seeking an independent third VAD practitioner if required, and including an independent psychiatrist to assess any concerns about capacity. An independent and external third‐party agency witnesses the patient's written declaration for VAD (www.dwdnsw.org.au/).

## Prisoner confidentiality

Confidentiality is extremely important for vulnerable prisoners; however, it is compromised in VAD by the obligation to share some health information with CSNSW, who decide on the operational responsibilities for the pathway (eg, escorting the patient to the hospital on the chosen date, risk assessment of the hospital location, organising staff to remain on duty over several shifts at the hospital, approving the use of least restrictive practices for the escort, and approving the access of visitors). Prisoners requesting VAD should be made aware of this and give their informed consent for sharing information.

## Workforce considerations

Another legally protected cornerstone of VAD is the notion of voluntary participation by practitioners and staff. In the short‐staffed correctional setting environment, relevant staff and their managers should receive education, training resources and support to ensure conscientious objectors can safely exclude themselves without obstructing the process, and still provide care for these patients.^13^ Fractured relationships and differing views on VAD by the prisoner's loved ones may be difficult to predict ahead of the final step (administration of VAD substance), when all parties come together for the first time. Managing family conflict can be stressful for the attending VAD practitioner, who may also experience othering and stigmatisation by the local hospital staff and subsequently by the media. Scrutiny of the case by the coroner, with the possibility of becoming a witness at a controversial inquest can be additionally stressful for involved staff, and access to psychological support is essential.

## Victims’ rights

The potential for victim distress is another concern, especially if terminally ill prisoners are considered for early release from custody to access VAD in the community. The NSW Charter of Victims’ Rights legislates that victims are notified if a perpetrator is released from custody, any relevant special conditions of their bail and if the perpetrator has died.^14^ Privacy laws restrict the sharing of health information about the cause of the prisoner's death (ie, by VAD); however, this may be inadvertently disclosed to victims through the coronial review process.

## Hypothetical patient journey

RT is a 56‐year‐old male prisoner with terminal cancer who is on a lengthy sentence. Despite optimal palliative care, RT is suffering a poor quality of life and has less than six months to live. His request for early release has been denied by the State Parole Authority. See Box for the journey of this hypothetical NSW prisoner on the VAD pathway.

Box 1 Hypothetical patient journey demonstrating voluntary assisted dying (VAD) pathway for a NSW prisoner*

**Table:** 

Step	Details
Initial enquiries	Prisoner RT approaches the health centre nurse about voluntary assisted dying (VAD). The nurse informs RT of their conscientious objection to VAD and contacts the palliative care social worker. Initial information about VAD is confidentially provided in the context of a discussion about all end‐of‐life options. He is directed to the easy‐read information on a tablet, which he accesses privately in his cell. He remains interested in VAD.
First request	The Justice Health and Forensic Mental Health Network (JHNSW) VAD practitioner, Dr ABC, meets confidentially with RT, who makes an official first request for VAD. Dr ABC accepts the request, becomes the coordinating VAD practitioner and completes the first request form. As for all decision‐making steps in the VAD pathway, this is documented in RT's health record and the form is submitted to the NSW VAD Board through the NSW Health VAD online portal within 5 business days.
First assessment	Dr ABC conducts the first (eligibility) assessment, providing a confidential interview to ensure RT is acting without coercion. RT's decision‐making capacity is presumed adequate (as for any health decision); however, if there are concerns, he is referred to a psychiatrist external to JHNSW for a capacity assessment. His palliative care and pain management and any associated anxiety or depression are properly addressed. RT is asked to provide consent for sharing essential information with Corrective Services NSW (CSNSW) and the local hospital, and he is made aware of the need for a coronial autopsy and review. Dr ABC completes and submits the first assessment report form and makes a referral to Dr XYZ to complete RT's second (eligibility) assessment.
Second assessment	To optimise independence, Dr XYZ is a VAD practitioner from a different JHNSW health care team in the prison who has not been caring for RT. After conducting the second assessment, Dr XYZ confirms RT's eligibility for VAD. Psychology and social work support is available for any patients assessed as ineligible for VAD.
Written declaration, final request and final review	RT completes a written declaration, formally requesting VAD. To avoid any potential conflict of interest, this declaration is made in the presence of two independent witnesses who are external to JHNSW or CSNSW. RT makes a final request for VAD to Dr ABC (following the legislated timeframes). Dr ABC completes the final review to ensure all steps have been followed in line with the *VAD Act 2022*.
Administration decision and family involvement	Dr ABC meets confidentially with RT to discuss options for the administration of the VAD substance. Due to his physical weakness, RT decides on intravenous practitioner‐administration (rather than oral self‐administration). RT wants his wife and children to be with him during the hospital admission for administration of the VAD substance. Dr ABC communicates this request to CSNSW who review and approve this under their visitor security protocol.
Substance authorisation and transfer	Following approval from the NSW VAD Board, Dr ABC prescribes the VAD substance and arranges for the NSW Health VAD Pharmacy Service to deliver the substance directly to Dr ABC at the hospital on the day of the VAD administration.
Preparation and coordination	A final multidisciplinary meeting is convened to ensure all stakeholders (JHNSW, CSNSW, the hospital, and coroner) are agreed on the hospital admission plan. The palliative care social worker liaises with CSNSW and the family to coordinate their travel plans. The family is briefed on the process, what to expect, and any additional psychosocial supports they require.
Substance administration	On the day of admission, Dr ABC places a secure cannula in RT's arm at the prison before his transfer to the hospital VAD bed. As per agreed process, the CSNSW escort will follow least restrictive practices. The family meets RT at the hospital and the admission is commenced early so that he can spend some time with his wife and children. RT and his family are aware that the bed is available for up to 8 hours (as for any VAD inpatient). Any delays are communicated to CSNSW so they can arrange their staffing and approve overtime. The VAD substance is delivered by the NSW Health VAD Pharmacy Service to Dr ABC, the VAD administering practitioner. The VAD pharmacist remains to witness the administration. At the agreed time, the substance is administered by Dr ABC who remains with RT and family until death and helps to facilitate the Coroner Police process.
Administration documentation and procedures	Following RT's death, the legal and medical documentation is completed, including the VAD practitioner administration form, the life extinct form, and reporting a death to the coroner form. A death certificate is not issued as this is provided by the coroner. The Coroner Police transport RT's body for the mandatory autopsy and coroner's review. Disposal of any unused VAD substance is managed by Dr ABC according to the legislative requirements and the NSW Health VAD Pharmacy Service.

* RT (hypothetical patient) is a 56‐year‐old male prisoner with terminal cancer who is on a lengthy sentence. Despite optimal palliative care, RT is suffering a poor quality of life and has less than six months to live. His request for early release has been denied by the State Parole Authority. The hypothetical patient journey is not an example of the complete VAD process, as detailed in the *NSW Voluntary assisted dying clinical practice handbook*.^13^

VAD has been provided to several prisoners in South Australia, despite SA's prison population being less than one quarter of the NSW prison population.^15^ To our knowledge, no other Australian state or territory has provided VAD to prisoners. The SA model is different to the NSW pathway; SA prisoners eligible for VAD attend consultations outside of the gaol with an independent third‐party provider and complete the final step (administration of VAD substance) at a local hospice. Most VAD deaths in SA are by self‐administration.^16^ This model has advantages by minimising the potential for coercion in the gaol environment and avoids the stigma and potential conflict between roles for local prison health teams. This independent third‐party model is unlikely to be implemented in NSW (where prisoner VAD deaths remain under the scrutiny of the coroner), but could be successfully adopted in the other states and territories (where, as in SA, they are only reported by exception).^17^

## Conclusion

The implementation of VAD in the NSW correctional setting creates tension between human rights, ethics and the law. The VAD pathway for NSW prisoners is not equivalent to community‐equivalent care; however, the interagency collaboration may in part address the disparities and provide a foundational model for other Australian correctional jurisdictions.

## Competing interests

No relevant disclosures.

## Provenance

Not commissioned; externally peer reviewed.

## Author contributions

Ahluwalia D: Project administration, resources, writing – original draft. Haysom L: Supervision, visualization, writing – review and editing.
